# Challenges in organizing effective oncology service: inter-European variability in the example of head and neck cancers

**DOI:** 10.1007/s00405-014-3197-x

**Published:** 2014-07-22

**Authors:** Julian Malicki, Wojciech Golusinski

**Affiliations:** 1 Department of Electroradiology, University of Medical Sciences, Poznan, Poland; 2 Department of Medical Physics, Greater Poland Cancer Centre, Poznan, Poland; 3 Department of Head and Neck Surgery, University of Medical Sciences, Poznan, Poland; 4 Greater Poland Cancer Centre, Poznan, Poland

## Abstract

The increasing worldwide burden of cancer makes it imperative that every country develop a comprehensive cancer control programme. In the past, cancer control in Central and Eastern Europe was inadequate, particularly when compared to many wealthier Western European countries. We analyse interregional differences in Europe to the approach to comprehensive cancer care, with a focus on head and neck squamous cell carcinoma using the case of Poland as a representative example. Due to national plans major improvements have been achieved in the field of prevention and in radiotherapy delivery having a measurable and positive impact on treatment outcomes. In head and neck cancers a notable move towards multidisciplinary approach has been made, combining surgery, radiotherapy and chemotherapy accompanied by rehabilitation and social support. In Poland and several other Eastern and Central European countries a shortage of physicians in the field of oncology was noted. The main conclusion is that the special plans are needed in Central and Eastern Europe or those existing must be extended for another decade to fulfil the EU requirement of providing all European citizens with equal access to quality cancer care.

## Introduction

The burden of cancer is increasing in European countries due to population ageing and lifestyle choices such as tobacco and alcohol use, physical inactivity, and poor diets [1–6]. As a result, demand for cancer care services has been growing as well. The challenge facing most countries is how to meet this rising demand as efficiently and effectively as possible.

These challenges are particularly relevant in cancer care, especially in times of limited budgets and increasingly expensive technology and drugs [7–16]. To reduce the costs associated with providing health care to a large population, it is essential to develop a comprehensive approach to cancer control. A substantial percentage of cancers could be prevented by applying existing cancer control knowledge to implement effective prevention and screening programmes and to undertake public health campaigns to promote physical activity and healthy diets. However, even though the effectiveness of such interventions is well-known, cancer control in many countries is woefully inadequate, and morbidity and mortality rates due to cancer are higher than they need be [3, 6, 17–25].

In the past, cancer control in Central and Eastern Europe was inadequate, particularly when compared to many wealthier Western European countries. However, in recent years, many countries in this region have made a concerted effort to improve cancer control and care. As a result, the health care systems in these countries have undergone several major reforms since the early 1990s to address their numerous shortcomings [26–29].

In the present article, we analyse interregional differences in Europe to the approach to comprehensive cancer care, with a focus on head and neck squamous cell carcinoma (HNSCC). The case of Poland is used as a representative example for Central and Eastern Europe, where gross national income (GNI) is significantly lower than in Western and Northern Europe.

## Cancer incidence in Poland and in the European Union

Historically, cancer care outcomes in Central and Eastern European countries have been below those reported in Western and Northern European countries [1, 4, 6, 23, 24, 30–32]. In 2003, for example, cancer survival rates in Poland were only 30 vs. 45 % in Western and Northern Europe [33–36]. Similarly, the Eurocare-3 study, which compared 19 European countries to assess associations between national income, investment in health care, and survival in patients diagnosed with cancer between 1990 and 1994 (and followed to 1999), found that all-cancer survival in Poland was lower than countries of similar wealth, in part due to relatively low spending on health care as a percentage of gross domestic product [37]. The EUROCARE-4, which assessed patients from 22 European countries diagnosed with cancer between 1995 and 1999 and followed to December 2003, reported that the mean European survival for HNSCC cancers was 48.9 %, with survival significantly lower in two countries: the United Kingdom (UK)-Northern Ireland (36.9 %) and Poland (37.9 %). This compares to higher survival rates found in Finland (59.7 %), Sweden (56.2 %) and Germany (61.2 %) [6]. Similarly, that same study found that survival for most solid cancers (whose prognosis depends on diagnostic stage), was lowest in the Czech Republic, Poland, and Slovenia. Publication of the EUROCARE results has encouraged many countries, among them the UK, Denmark, and Poland, to develop a national cancer plan to improve outcomes.

## HNSCC

Head and neck squamous cell carcinoma is a significant component of the global burden of cancer. Worldwide, more than 600,000 patients are diagnosed each year with HNSCC, accounting for 6 % of all cancer cases. Studies have shown that heavy intake of alcoholic beverages is associated with nutrient deficiency, which appears to contribute independently to oral carcinogenesis [5]. In the last two decades, a slight decrease has been noted in the overall incidence of head and neck cancer, laryngeal cancer in particular. In contrast, however, a significant increase in cancers of the oropharynx and oral cavity has been detected [23, 38]. Oral cancer is particularly high among men, and more common in developing than developed countries. Sharp increases in the incidence rates of oropharyngeal cancers have been noted for several countries and regions, including Denmark, France, Germany, Scotland, and Central and Eastern Europe [5].

According to the Central Statistical Office in Poland, 5,645 head and neck cancer deaths were registered in Poland in 2010 (4,430 men, 1,215 women), constituting an increase of 11 % compared to 1999. In Poland, H&N cancer accounts for 8.5 % of cancer deaths in men and 3 % in women. Patients age 55+ accounted for most of those cancer-related deaths (80 and 85 %, respectively, in men and women). The raw mortality rate also increased with age. The male to female incidence ratio of 1.8 shows that the relative risk of developing H&N cancer is generally higher in men, as is the relative risk of death (M/F = 3.8), particularly in the 55–59 age group in which the M/F ratio rises to 5.8. In most categories, the morbidity/mortality (MM) ratio is higher than 1, with the exceptions of men over age 80 and women over age 85. Of particular note is the 30–34 age group, in which the MM ratio for women is almost 20× higher than for men [35, 39–41].

Researchers have identified the human papilloma virus (HPV) as the causative factor for a subset of HNSCC tumours [42–44], particularly HPV-16, which is especially common in otopharyngeal tumours. Fortunately, HPV-associated tumours have better clinical outcomes [43]. As in many other cancers, oral cancer is preventable by minimizing or eliminating risk factors. Prevention of HIV infection will also reduce the incidence of HIV/AIDS-related cancers such as Kaposi’s sarcoma and lymphoma. In 2007, the World Health Assembly passed a resolution (the WHO Global Oral Health Programme; WHA60 A16) urging member states to ensure that prevention be included as a major component of cancer control programmes, along with the involvement of oral health professionals or primary health care staff with relevant training in oral health, in detection, early diagnosis, and treatment [5].

Head and neck cancers are difficult to treat due to their heterogeneity, location near sensitive organs, and the fact that treatment is nearly always multidisciplinary [16, 38, 41, 45–47]. Depending on the site and stage of the cancer, treatment may consist of surgery, radiotherapy, chemotherapy or a combination thereof, accompanied by rehabilitation and social support [2, 45]. For advanced cases, there has been a shift from surgical treatment towards chemoradiotherapy protocols (especially concomitant chemotherapy and radiotherapy). Systemic therapies (chemotherapy and targeted molecular agents) have been successfully integrated into potentially curative treatment of locally advanced HNSCC [15, 39, 48, 49]. Major improvements have been achieved in radiotherapy delivery [11, 29, 50–52], and in Poland a recent study found that investments in more modern radiotherapy equipment had a measurable and positive impact on treatment outcomes [27, 53, 54]. In deciding which treatment strategy would be suitable for an individual patient, important considerations include expected functional outcomes, ability to tolerate treatment, and comorbid illnesses. Collaboration amongst many specialties is the key for optimal assessment and decision-making in the management of HNSCC [38, 46].

## Network of comprehensive cancer centres in Poland

In Poland, the largest and most populous country in the region, a major cancer control initiative was undertaken in 2005 with the implementation of the National Program against Cancer Diseases (NPACD) [33]. The main aim of the NPACD was to combat the alarming and increasing incidence of cancer. To that end, the government allocated approximately €700 million (PLN 250 million/year) in funding for the years 2005–2015. In addition to the aforementioned goal of preventing cancer, the NPACD also sought to bring Polish cancer care up to European standards for early cancer diagnosis and treatment, to accelerate the transfer of the latest research findings from lab to bed, and to monitor the efficacy of cancer control activities in Poland.

The current medical care system in Poland is an obligatory public health insurance system. All individuals covered by general health insurance receive free health services by private or public health care providers. Contracts are awarded on a regional basis directly by one of the 16 branches of the National Health Fund (NHF) and are signed for 5 years while reimbursement is negotiated annually. Patients also have the right to choose their health care provider, which can be public (mainly hospitals) or private (individual or group medical practices or regular commercial entities). Poland joined the EU more than 10 years ago, and the consequent increase in cross-border patient and staff mobility has increased expectations in Poland, not only for improved treatment results and greater comfort in the hospital setting [55].

Since 2009, due to insufficient funds, the NHF has had difficulties fully reimbursing hospitals. At present, private hospitals compete with public hospitals on an equal basis for NHF contracts. However, some have questioned this rule because even though both private and public hospitals provide health services to patients at no cost to the patient (i.e., reimbursed by the NHF), private hospitals are also allowed to treat and charge private patients outside the NHF. Public hospitals, in contrast, are not allowed to have private patients, with only a few exceptions (e.g., procedures not covered by the NHF). This is a major handicap to public providers when the NHF contract is fulfilled before the end of the year: private providers have the option to close or limit service while public ones are forced to continue treating patients, even though they will not receive any additional funds for doing so, thus worsening their financial condition.

In recent years, the NPACD has provided substantial funds for investment in new equipment, to develop screening and prevention programmes (including genetic screening), to improve cancer registries, and to carry out epidemiological studies. Other sources of funds include the National Science Centre and National Centre for Research and Development, both of which support research projects through grants, plus local sources of funding that can be used for investment and prevention/screening.

The existing cancer network consists of comprehensive cancer centres located in each of the 16 provinces in Poland. Universities also provide cancer care at their teaching hospitals through small clinics within these hospitals. Such clinics are legally part of a large multidisciplinary teaching hospital, and thus have no financial autonomy. In the authors’ opinion, some changes in the law are needed to enable existing cancer centres to become more involved in academics, either by granting them status as research institutes or by allowing them to enter the university as stand-alone centres. At the very least, it would be important to avoid forcing such centres to be “diluted” as part of general medicine hospitals.

An important issue in Poland—and several other Eastern and Central European countries—is a shortage of physicians in the field of oncology [27, 56]. As in many other countries, the discipline of oncology in Poland is subdivided into several different specialities, including oncological surgery (although general surgeons can also operate on cancer patients), radiation oncology, and medical oncology. In contrast, in some countries, notably the Scandinavian countries and the United Kingdom, the specialties of medical and radiation oncology are combined. At present, there is much debate about the appropriate competencies of these various specialities, particularly with regards to prescribing oral anticancer drugs [7, 57].

One of the drivers of the shortage of specialists in radiation oncology, in Poland and other countries, is that, compared to other specialities, fewer students choose this field for the simple reason that it is introduced towards the latter part of medical studies, when many students have already chosen their preferred specialities [53, 57]. Since 2009, the NPACD has required that all universities in Poland provide at least 15 contact hours of basic oncology in the 3rd year curriculum of the degree in medicine, and 60 contact hours of oncology in the 6th year. Finally, another challenge relates to awareness and training of university students who are studying fields (other than medicine) in which an understanding of oncology is—or should be—important. Such fields include medical physics, physiotherapy, nursing, public health, pharmacy, biotechnology, and many others. The process of incorporating oncology into the curriculum, both as an undergraduate course for future physicians and for other medical disciplines, is ongoing [27, 57, 58].

## Conclusion

As we have seen in the case of Poland, Eastern and Central European countries have undertaken comprehensive changes and increased spending to improve their cancer control and treatment efforts in recent decades. The result is a much improved health care system with comprehensive cancer control efforts and improved technology and training.

However, unlike Poland, not all countries in the European Union have the funds to invest in more modern equipment and facilities, particularly in Central and Eastern Europe. However, countries with fewer resources can still greatly improve health care outcomes. Research by the WHO and other international bodies has shown that the most cost-effective way to improve outcomes and to reduce the burden of cancer is through coordinated prevention and early detection efforts. This approach offers countries a low cost but effective way to control cancer, and it has been successfully implemented in Poland. At the same time, however, prevention and detection also require well-run national and regional cancer registries to keep track of changes, and so it is imperative that countries seeking to improve cancer control implement a coordinated approach to preventing and treating cancer.
